# Higher cardiovascular disease risks in people living with HIV: A systematic review and meta-analysis

**DOI:** 10.7189/jogh.14.04078

**Published:** 2024-04-26

**Authors:** San Zhu, Wenjing Wang, Jiaze He, Wenshan Duan, Xiaoran Ma, Honglin Guan, Yaxin Wu, Sibo Li, Yanbing Li, Tian Tian, Wenjun Kong, Dongxia Wu, Tong Zhang, Xiaojie Huang

**Affiliations:** 1 Clinical and Research Center for Infectious Diseases, Beijing Youan Hospital, Capital Medical University, Beijing, PR China; 2 West China School of Medicine, Sichuan University, Chengdu, Sichuan, PR China; 3 Tianjin University, Tianjin, PR China; 4 Hematology Department, The First Affiliated Hospital of China Medical University, China Medical University, Shenyang, PR China; 5 Cardiovascular Department, Beijing Youan Hospital, Capital Medical University, Beijing, PR China; 6 Department of Ophthalmology, Beijing Youan Hospital, Capital Medical University, Beijing, PR China

## Abstract

**Background:**

The prognosis of AIDS after active antiretroviral therapy (ART) and the quality of life of people living with HIV (PLWH) are both affected by non-AIDS-related diseases such as cardiovascular disease (CVD). However, the specific risk ratios between PLWH and individuals negative for HIV are poorly understood. We aimed to systematically review and investigate the CVD risk factors associated with HIV.

**Methods:**

We searched PubMed, Embase, Web of Science, and Cochrane Library databases between 1 January 2015, and 12 May 2023 for articles reported the prevalence and risk factors of CVD such as hypertension, dyslipidaemia, coronary artery disease (CAD), and myocardial infarction (MI). Due to the high heterogeneity, we used a random-effects model to analyse the data. All statistical analyses were performed using Stata/MP 17.0 with 95% confidence intervals (CIs).

**Results:**

We analysed 31 eligible studies including 312 913 PLWH. People living with HIV had higher risks of dyslipidaemia (hazard ratio (HR) = 1.53; 95% CI = 1.29, 1.82), CAD (HR = 1.37; 95% CI = 1.24, 1.51), and MI (HR = 1.47; 95% CI = 1.28, 1.68) compared to individuals without HIV. However, there were no significant differences in the prevalence of hypertension between groups (HR = 1.17; 95% CI = 0.97, 1.41). Subgroup analysis revealed that men with HIV, PLWH who smoked and the elderly PLWH had a high prevalence of CVD. Moreover, the disease prevalence patterns varied among regions. In the USA and Europe, for instance, some HRs for CVD were higher than in other regions. Active ART initiation after 2015 appears to have a lower risk of CVD (hypertension, hyperlipidaemia, CAD). All outcomes under analysis showed significant heterogeneity (*I^2^*>70%, *P* < 0.001), which the available study-level variables could only partially account for.

**Conclusions:**

People living with HIV had a higher CVD risk than the general population; thus, CVD prevention in PLWH requires further attention. Rapid initiation of ART may reduce the incidence of CVD in PLWH. For timely screening of CVD high-risk individuals and thorough disease management to prevent CVD, further studies are required to evaluate the risk factors for CVD among PLWH, such as age, region, etc.

**Registration:**

PROSPERO (CRD42021255508).

The widespread use of antiretroviral therapy (ART) has risen the life expectancy of patients with AIDS [1]. Simultaneously, the number of elderly people living with HIV (PLWH) is rising and expected to continue. The proportion of PLWH worldwide aged ≥50 years or was expected to increase from 21% at the end of 2019 to 73% by 2030, according to the latest estimates by the United Nations Programme on HIV/AIDS [2]. Thus, the prognosis and quality of life of PLWH will be impacted by AIDS and various non-AIDS-related diseases such as cardiovascular disease (CVD) [3,4]. The 2021 United States Department of Health and Human Services antiviral treatment guidelines recommended the close monitoring of cardiovascular health in elderly PLWH [5]. Recent studies have demonstrated the pooled prevalence of coronary artery disease (CAD) [6,7], cardiac dysfunction [8,9], dyslipidaemia [10], and hypertension in PLWH [11]. They have indicated that in regions with established ART, CVD is the primary cause of mortality [12]. Various studies have highlighted subclinical CAD as a marker of cardiovascular clinical events [13], further highlighting the importance of reducing the relative risk of myocardial infarction (MI) in PLWH [14,15]. However, the supposedly higher CVD risk in PLWH clinically has not been specifically and comprehensively illustrated; thus, this complication is likely underestimated in the vulnerable population [16].

In 2015, the World Health Organization recommended immediate initiation of ART for PLWH with uncertain CD4^+^T cell count [17]. However, clinical monitoring and treatment of CVD in PLWH have not received sufficient attention. Hence, a more comprehensive assessment of CVD burden in PLWH would aid in identifying the specific needs for reducing CVD risk factors.

The present systematic review and meta-analysis assessed the pooled effect size (ES) for specific CVD risk in PLWH compared with that in normal individuals, according to different factors. Considering the broad nature of CVD, we focused on four representative conditions: hypertension, dyslipidaemia, CAD, and MI.

## METHODS

The study was registered in the International Prospective Register of Systematic Reviews (PROSPERO) [18] (CRD42021255508) and reported according to the Preferred Reporting Items for Systematic Reviews and Meta-Analyses (PRISMA) statement [19], which can be found in the PRISMA checklist (**Online Supplementary Document**).

### Data sources and search strategy

For studies reporting the prevalence and risk of CVD in PLWH published between 1 January 2015, and 12 May 2023, we searched the PubMed, Embase, Web of Science, and Cochrane Library databases using following subjects and keywords: ‘coronary heart disease?’, ‘atherosclerosis’, ‘myocardial infarction’, ‘dyslipidaemia’, ‘hyperlipidaemia’, ‘hypertension’, ‘ART’, ‘HIV’, ‘hazard ratio’, ‘odds ratio’, and ‘risk ratio’. To discover new pertinent articles while writing the manuscript, automatic PubMed literature alerts were set up. We also used a back search of reference lists of eligible studies and relevant review papers to ensure the comprehensive search (Tables S1–S2 in **Online Supplementary Document**) provide specifics about the search strategy.

### Inclusion and exclusion criteria

Inclusion criteria were studies (1) published from 2015 onward that reflected the current state of HIV treatment and disease management; (2) published in English; (3) that presented the odds ratio (OR), or hazard ratio (HR), or relative risk (RR) of PLWH and the general population or the incidence of MI, CAD, hypertension, or dyslipidaemia; (4) that stratified the results by region, age, follow-up duration, CD4^+^T cell count, plasma viral load, and ART use; and (5) that used standardised definitions for the diseases presented in Table S3–S4 in **Online Supplementary Document**.

To minimise the consequences of selective reporting, unpublished reports and conference abstracts were eliminated. Furthermore, studies using intermediate, surrogate, or CVD biomarker outcomes as well as those involving animals, children, or pregnant women were excluded. Studies examining no systemic hypertension (such as pulmonary and portal hypertension) or those recruiting patients with conditions linked to hypertension (such as kidney failure and heart disease) were omitted. Table S5 in **Online Supplementary Document** details these criteria.

### Data sources and search strategy

We screened and managed literature with EndNote X9. Three reviewers (S Zhu, WJ Wang, and JZ He) independently reviewed the titles and abstracts to assess their eligibility. We resolved differences through discussion; when necessary, we invited a fourth investigator (WS Duan) to help reach a consensus.

We gathered all data on the prevalence and risk factors for hypertension, dyslipidaemia, CAD, and MI among PLWH. We also developed a standardised table to extract data such as authors, study year, sample size, study demographic characteristics, and outcome measures. A third investigator (S Zhu) compiled the results after two reviewers (WJ Wang and JZ He) independently extracted the data.

### Study selection and data extraction

We screened and managed literature with EndNote X9. Three reviewers (S Zhu, WJ Wang, and JZ He) independently reviewed the titles and abstracts to assess their eligibility. We resolved differences through discussion; when necessary, we invited a fourth investigator (WS Duan) to help reach a consensus.

We gathered all data on the prevalence and risk factors for hypertension, dyslipidaemia, CAD, and MI among PLWH. We also developed a standardised table to extract data such as authors, study year, sample size, study demographic characteristics, and outcome measures. A third investigator (S Zhu) compiled the results after two reviewers (WJ Wang and JZ He) independently extracted the data.

### Risk of bias analysis

The Newcastle-Ottawa Scale was used to evaluate cross-sectional and cohort studies [20]. Overall, scores of 7–9, 6, and 0–5 were considered high, medium, and low quality, respectively. A third researcher (S Zhu) resolved conflicts after two researchers (WJ Wang and SB Li) independently screened and extracted the data. Table S6 in **Online Supplementary Document** shows the results of the quality evaluation.

### Statistical analysis

The statistical analyses and 95% confidence intervals (CIs) were produced using Stata/MP 17.0 (StataCorp, College Station, TX, USA). We pooled the prevalence of various CVDs (hypertension, dyslipidaemia, CAD, and MI). The risk of CVD (HR) in PLWH compared with HIV-uninfected people was summarised and presented. In the search, we included ‘HR’, ‘RR’ and ‘OR’ for the purpose of including relevant studies as comprehensively as possible. In general, the OR and the RR cannot be substituted for each other. However, the disease (outcome) studied is relatively rare, and when its occurrence rate is less than 10%, OR can be used as an approximate estimate of RR [21]. For the conversion of RR, HR and OR, we used OR = ((1-P_0_)*RR)/(1-P_0_*RR) and RR ≈ (1-e^HR*ln(1-P0)^)/P_0_ in the data analysis. P_0_ refers to the incidence of the control groups [22].

Using *I^2^* statistic, heterogeneity between studies was analysed. *I^2^* values of 0–40%, 30–60%, 50–90%, and 75–100% indicated low, medium, substantial, and high heterogeneity. A fixed- or random-effects model was utilised for data synthesis, depending on the heterogeneity. To investigate the origins of heterogeneity, subgroup analyses were conducted to explore factors affecting the risk of CVD. Analyses were stratified by age (≤50 or >50 years), region, race, follow-up duration, and proportions of males and smokers. Additionally, correlations were examined between important HIV-related indicators (time since HIV diagnosis, ART duration, and CD4^+^T cell count) and the HR of CVD to assess the impact of immune factors on CVD risk. We pooled the data using adjusted risk estimates to minimise bias.

We used funnel plots to assess publication bias and also performed the sensitivity analysis by excluding studies from the meta-analysis one by one. The threshold for statistical significance was set at *P* < 0.05.

## RESULTS

### Study characteristics and quality assessment

**Figure 1:**
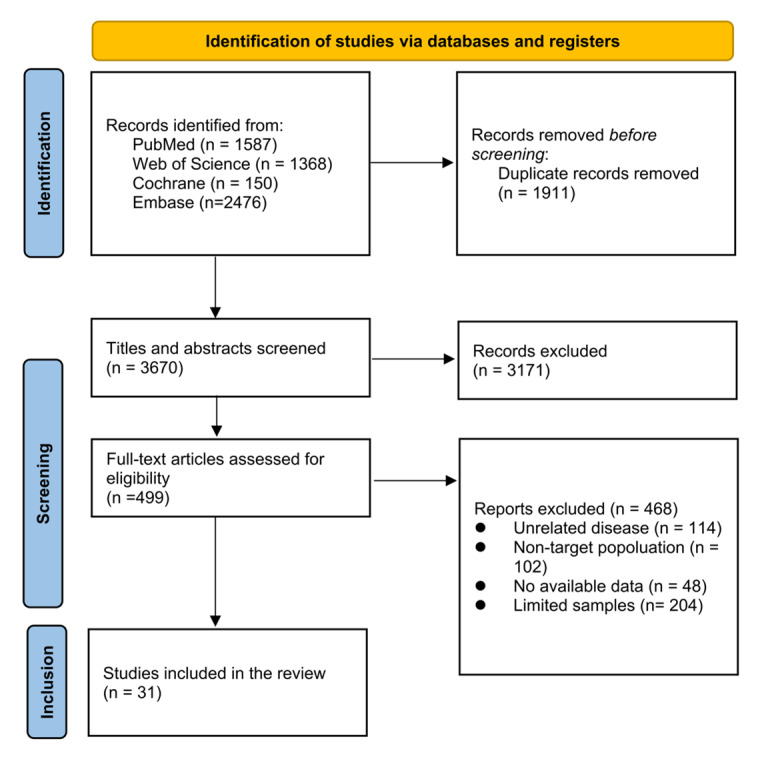
Flowchart of the inclusion of studies.

The comprehensive database search identified 5581 records. After assessing titles and abstracts and removing duplicates, 499 articles were left for full-text evaluation. During the full-text screening, 114 articles reporting none the four chosen CVD were excluded, 306 articles were excluded because the subjects did not match the inclusion criteria, and 48 articles were excluded because the required data could not be obtained. Finally, a total of 31 studies [23–53] meeting the inclusion criteria were included, of which 17 were cross-sectional [23,25,32,36,43,45–53] and 14 were prospective cohort studies [24,26–31,33–35,37–42,44] (**Figure 1**).

These studies involved 40 551 855 participants, 312 913 of whom were PLWH. Over 80% of patients were men. The majority of these studies were carried out in North America and Europe. The detailed baseline characteristics of the trials and enrolled individuals were listed in **Table 1**.

**Table 1:** Characteristics of the included studies

Study	Country		Study period	Total sample (HIV+/%)	Male, n (%)*	Mean (SD) or median (IQR) age, years	Tobacco use, n (%)*	Years of HIV infection, median (IQR), y	ART users, n (%)	Mean years of ART median (IQR), y	Current CD4^+^T count, cells/μL median (IQR) (Current CD4 group >500, %)	Nadir CD4^+^T cell count, median (IQR)	On ART, undetectable HIV viral load, n (%) (<x copies/ml)
**Hypertension**	
Hasse B et al. (2015)	Switzerland		2003–2013	74 291 (3230 / 4.3)	36 266 (48.8)	58.0	(36.5)	14.0 (9.5, 19.0)	(75.3)	3.5	572.0 (420.0, 754.0)	172.0 (70.0, 259.0)	NR
Tripathi A et al. (2015)	US		1994–2011	13 632 (6816 / 50.0)	3863 (56.7)	38.0 (31.0, 46.0)	1994 (14.6)	NR	(80.0)	NR	NR	NR	NR
Friedman EE et al. (2016)	US		2006–2009	29 060 418 (24 735 / 0.09)	14 419 (58.3)	71.1	NR	NR	NR	NR	NR	NR	NR
van Zoest RA et al. (2016)	The Netherlands		2010–2012	1044 (527 / 50.5)	467 (88.6)	52.9 (48.3, 59.6)	353 (67.0)	12.2 (6.6, 17.2)	499 (94.7)	NR	570.0 (435.0, 745.0)	180.0 (80.0, 260.0)	493 (99.6%) (<200)
Gelpi M et al. (2018)	Copenhagen		2015–2016	13 260 (1099 / 8.3)	937 (85.3)	50.1 (42.8, 58.0)	691 (62.8)	13.7 (6.9, 21.3)	1078 (98.4)	10.5 (5.2, 17.3)	690.0 (77.5%)	CD4 nadir <200, % (n):42.0 (450.0)	1030 (94.7%) (<50)
Mayer KH et al. (2018)	US		2006–2016	239 849 (12 837 / 5.4)	8984 (70.0)	40.3 (11.8)	6176 (48.1)	NR	NR	NR	572.0	–	(73.2%) (undetectable)
Rücker SCM et al. (2018)	Chiradzulu, Malawi		2015–2016	735 (379 / 51.6)	102 (26.9)	47.0 (42.0–52.0)	9 (2.4)	NR	NR	NR	NR	NR	(92.7%) (<1000)
Manne-Goehler J et al. (2019)	South Africa		2014–2015	4547 (1035 / 22.8)	168 (55.8)	55.9	NR	NR	(70.9)	NR	NR	NR	551 (75.1%) (undetectable)
Yang HY et al. (2019)	US		2007–2016	9 141 867 (24 636 / 0.27)	74	59.2	(21.5)	NR	NR	NR	NR	NR	NR
Russell EAB et al. (2020)	Canada		2013–2017	289 (156 / 54.0)	0 (0.0)	43.5 (36.8, 50.9)	70 (44.8)	15.7 (10.9, 19.9)	144 (92.3)	NR	570.0 (335.0, 725.0)	190.0 (88.0, 289.0)	NR
Touloumi G et al. (2020)	Greece		2012–2016	10 659 (5839 / 54.8)	4984 (85.4)	41.6 (34.0, 49.7)	NR	6.6 (2.8, 13.2)	5025 (86.1)	6.4 (2.9, 13.3)	606.0 (416, 819)	NR	4286 (74.7%) (<50)
Xu X et al. (2021)	Taizhou (CHART), China		2017	4416 (1472 / 33.3)	1113 (75.6)	43.9 ± 14.1	556 (37.8)	2.7 (0.7, 5.4)	1344 (91.3)	2.2 (0.6, 4.3)	39.40%	NR	1304 (88.6%) (<200)
Enriquez R et al. (2022)	Uganda		2016–2018	1968 (990 / 50.3)	36.5	41.0 (4.0)	9	NR	NR	NR	NR	NR	NR
Gooden TE et al. (2022)	UK		2000–2020	46 049 (9233 / 20.1)	6061 (65.6)	NR	4042 (43.8)	NR	NR	NR	NR	NR	NR
Jones BI et al. (2022)	UK		1988–2017	8835 (2945 / 33.3)	1941 (65.9)	39.1 (12.7)	NR	NR	NR	NR	NR	NR	NR
Mogaka JN et al. (2022)	Kenya		2017–2018	598 (300 / 50.1)	150 (50)	HIV+: 45.0 (39.5, 53.0); HIV-: 40.0 (IQR = 31.0, 55.0)	39 (13.0)	NR	300 (100.0)	NR	NR	NR	213 (71.0%) (undetectable)
Morales DR et al. (2022)	UK		As of 30 November 2015	942 077 (964 / 0.1)	595 (61.7)	NR	NR	NR	NR	NR	NR	NR	NR
**Dyslipidaemia**	
Friedman EE et al. (2016)	US		2006–2009	29 060 418 (24 735 / 0.09)	14 419 (58.3)	71.1	NR	NR	NR	NR	NR	NR	NR
Gelpi M et al. (2018)	Copenhagen		2015–2016	13 260 (1099 / 8.3)	937 (85.3)	50.1 (42.8,58.0)	691 (62.8)	13.7 (6.9, 21.3)	1078 (98.4)	10.5 (5.2, 17.3)	690.0 (77.5%)	CD4 nadir**†**<200, % (n): 42.0 (450.0)	1030 (94.7%) (<50)
Mayer KH et al. (2018)	US		2006–2016	239 849 (12 837 / 5.4)	8984 (70.0)	40.3 (11.8)	6176 (48.1)	NR	NR	NR	572.0	-	(73.2%) (undetectable)
Rücker SCM et al. (2018)	Chiradzulu, Malawi		2015–2016	735 (379 / 51.6)	102 (26.9)	47.0 (42.0, 52.0)	9 (2.4)	NR	NR	NR	NR	NR	(92.7%) (<1000)
Masyuko SJ et al. (2020)	Western Kenya		2017–2018	598 (300 / 50.2)	150 (50.0)	45.0 (39.5, 53.0)	NR	9.0 (5.0, 11.0)	300 (100.0)	8.0 (4.0, 10.0)	512.0 (364.0, 666.0)	369.0 (215.0, 563.0)	235 (78.9%) (<50); 285 (95.6%) (<1000)
Russell EAB et al. (2020)	Canada		2013–2017	289 (156 / 54.0)	0 (0.0)	43.5 (36.8, 50.9)	70 (44.8)	15.7 (10.9, 19.9)	144 (92.3)	NR	570.0 (335.0, 725.0)	190.0 (88.0, 289.0)	NR
Touloumi G et al. (2020)	Greece		2012–2016	10 659 (5839 / 54.8)	4984 (85.4)	41.6 (34.0, 49.7)	NR	6.6 (2.8, 13.2)	5025 (86.1)	6.4 (2.9, 13.3)	606.0 (416.0, 819.0)	NR	4286 (74.7%) (<50)
Tilahun H et al. (2021)	Sub-Saharan Africa		2017–2018	564 (287 / 50.9)	144 (50)	≥30.0	38 (13.0)	9.0 (5.0, 11.0)	287 (100.0)	8.0 (4.0, 10.0)	512.0 (364.0, 666.0)	365.0 (213.0, 571.0)	275 (96.0%) (<1000)
Enriquez R et al. (2022)	Uganda		2016–2018	1968 (990 / 50.3)	360 (36.4)	41.0 (4.0)	85 (9.0)	NR	NR	NR	NR	NR	NR
Morales DR et al. (2022)	UK		As of November 30, 2015	942 077 (964 / 0.1)	595 (61.7)	NR	NR	NR	NR	NR	NR	NR	NR
**CAD**	
Chow D et al. (2015)	US		NR	2833 (100 / 3.5)	100 (100.0)	53.92 (6.71)	63 (63.0)	193.73 (91.41)	100 (100.0)	NR	497.20 (252.75)	149.26 (152.78)	84 (84.0%) (undetectable <48)
Kingsley LA et al. (2015)	US		2004–2010	825 (541 / 65.6)	541 (100.0)	49.2 (6.3)	403 (74.5)	NR	478 (88.4)	6.6 (2.6)	519.0 (360.0, 704.0)	278.0 (156.0, 391.0)	331 (60.7%) (undetectable)
Miller PE et al. (2015)	US		2010–2013	764 (453 / 59.2)	453 (100.0)	52.0 ± 6.5	(73.9)	NR	88.6	9.5 (6.4, 12.4)	599.0 (426.0, 774.0)	293.0 (178.0, 416.0)	(80.4%) (<50)
Mayer KH et al. (2018)	US		2006–2016	239 849 (12 837 / 5.4)	8984 (70.0)	40.3 (11.8)	6176 (48.1)	NR	NR	NR	572.0	–	(73.2%) (undetectable)
Yang HY et al. (2019)	US		2007–2016	9 141 867 (24 636 / 0.27)	74	59.2	(21.5)	NR	NR	NR	NR	NR	NR
Rosenson RS et al. (2020)	US		2011–2016	412 130 (82 426 / 20.0)	69 137 (83.9)	≥19.0	5640 (6.8)	NR	79 095 (96.0)	NR	NR	NR	NR
Tarr PE et al. (2020)	Switzerland		2013–2019	430 (340 / 79.1)	367 (85.3)	52.0 (49.0, 57.0)	151 (35.3)	15.1 (6.6, 21.8)	397 (92.8)	11.6 (5.3, 17.7)	600.0 (447.0, 752.0)	190.0 (90.0, 282.0)	374 (87.4%) (undetectable)
**MI**	
Badejo OA et al. (2015)	US		2009–2015	73 398 (18 289 / 24.9)	97	48.0 (±9.0)	NR	NR	NR	NR	29.64%	NR	NR
Klein DB et al. (2015)	US		1996–2011	282 368 (24 768 / 8.8)	91	41.0	45	NR	90	NR	605.0	303.0	(88.0%) (<500)
Paisible AL et al. (2015)	US		2003–2009	81 322 (26 831 / 33.0)	97.4	46.0	(53.1)	NR	51	NR	295.0	NR	(44.5%) (<500)
Rasmussen LD et al. (2015)	Denmark		1995–2013	16 165 (3233 / 20.0)	2548 (78.8)	44.6 (40.0, 52.5)	2125 (65.7)	5.8 (3.1, 8.6)	2497 (77.2)	7.2 (2.9, 12.2)	470.0 (320.0, 635.0)	NR	2296 (71.0%) (<400)
Drozd DR et al. (2017)	North America		1995–2014	28 912 (28 912 / 100.0)	23 284 (80.5)	≥18.0	22 130 (76.5)	NR	14 312 (49.5)	NR	31.3%	NR	8774 (30.3%) (<400)
Mayer KH et al. (2018)	US		2006–2016	239 849 (12 837 / 5.4)	8984 (70.0)	40.3 (11.8)	6176 (48.1)	NR	NR	NR	572.0	–	(73.2%) (undetectable)
Alonso A et al. (2019)	Michigan		2009-2015	79 100 (19 798 / 25.0)	15 972 (81.0)	43.0 (13.0)	1940 (9.8)	NR	NR	NR	NR	NR	NR
Masiá M et al. (2019)	Spain		2004–2015	9712 (9712 / 100.0)	7986 (82.2)	36.2	2379 / 4798 (49.6)	NR	9712 (100.0)	NR	NR	274.0 (145.0, 399.0)	NR
Rosenson RS et al. (2020)	US		2011–2016	412 130 (82 426 / 20.0)	69 137 (83.9)	≥19.0	5640 (6.8)	NR	79 095 (96.0)	NR	NR	NR	NR
Gooden TE et al. (2022)	UK		2000–2020	46 049(9233 / 20.1)	6061 (65.6)	NR	4042 (43.8)	NR	NR	NR	NR	NR	NR

ART – antiretroviral therapy, CAD – coronary artery disease, IQR – interquartile range, MI – myocardial infarction, NR – not reported, SD – standard deviation

*The proportion of indicators in people living with HIV.

†CD4 nadir means the lowest point to which CD4 counts have fallen during the course of the study.

Two medium-quality studies and 29 low-quality studies were detected in the risk of bias analysis, indicating good quality (Table S6 in the **Online Supplementary Document**). The funnel plots did not reveal statistically significant indication of publication bias (Figure S1 in the **Online Supplementary Document**).

### Hypertension

**Figure 2:**
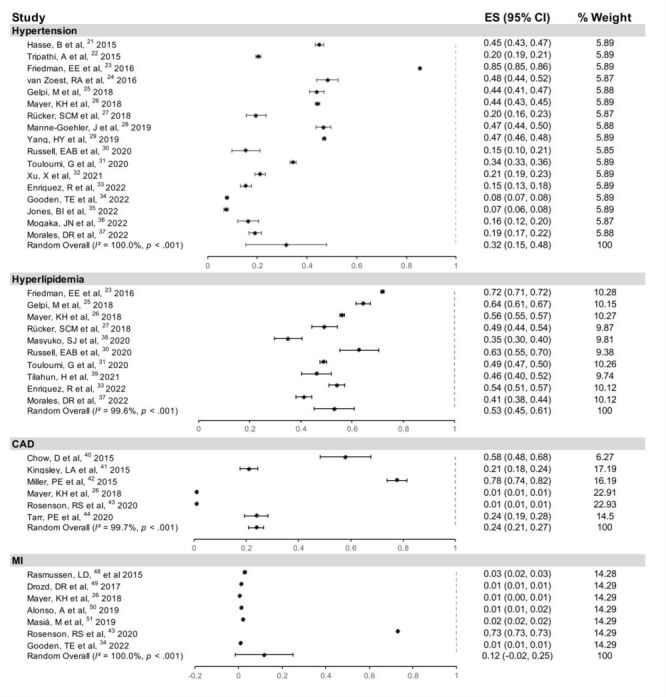
Forest plots of the different prevalence levels of CAD, MI, dyslipidaemia, and hypertension in PLWH. CAD – coronary artery disease, CI – confidence interval, MI – myocardial infarction, PLWH – people living with HIV

**Figure 3:**
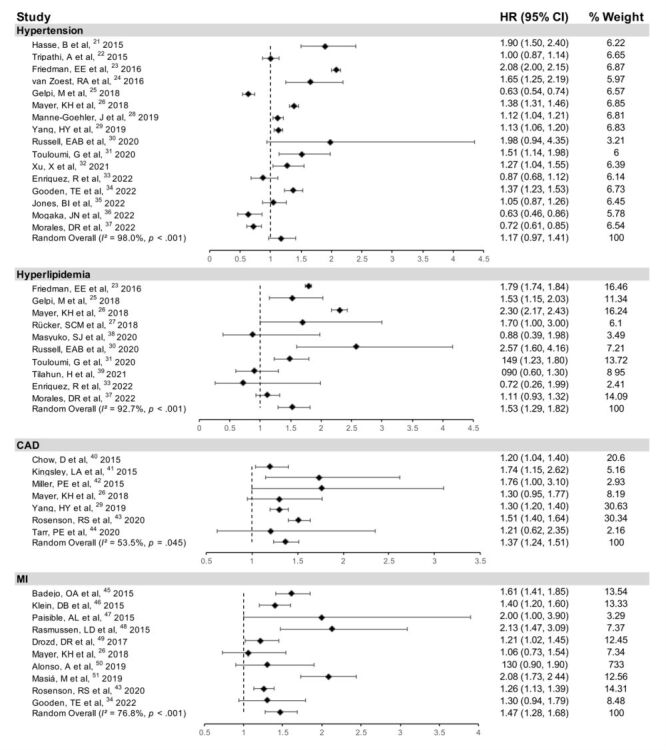
Pooled HRs of hypertension, dyslipidaemia, CAD, and MI in PLWH compared to the general population. CAD – coronary artery disease, CI – confidence interval, HR – hazard ratio, MI – myocardial infarction, PLWH – people living with HIV

The hypertension analysis included 17 studies [23–39] involving 39 562 734 patients. **Figure 2** showed that the prevalence of hypertension in PLWH was 32%. Comparable to general population, the HR of PLWH having hypertension was 1.17 (95% CI = 0.97, 1.41, *I^2^* = 98%) (**Figure 3**). Subgroup analysis revealed a slightly increased in hypertension risk with age (≥50: HR = 1.36, 95% CI = 0.97, 1.91; <50: HR = 1.08, 95% CI = 0.88, 1.31) (Figure S2 in the **Online Supplementary Document**). North America had higher risk in hypertension (HR = 1.40; 95% CI = 1.02, 1.92) compared with other regions (Europe, Africa, and Asia) (Figure S3 in the **Online Supplementary Document**). The HR was 1.08 (95% CI = 0.94, 1.25) after 2015 compared with 1.36 (95% CI = 0.85, 2.18) before 2015, based on the follow-up duration (Figure S4 in the **Online Supplementary Document**). The proportions of men and smokers did not differ significantly (Figures S5–6 in the **Online Supplementary Document**). The racial-ethnic differences also did not affect the risk of hypertension (Figure S7 in the **Online Supplementary Document**). No correlation was observed between the HR of hypertension in PLWH and time since HIV diagnosis, ART duration and CD4^+^T cell count (Figure S8 in the **Online Supplementary Document**).

### Dyslipidaemia

There were 30 043 828 patients in 10 studies [25,27–29,32,33,35,39–41]. In PLWH, dyslipidaemia was seen in 53% of cases (**Figure 2**). Compared to the general population, HR for risk of dyslipidaemia in PLWH was 1.53 (95% CI = 1.29, 1.82, *I^2^* = 92.7%) (**Figure 3**). In subgroup analysis, PLWH aged ≥50 years had an HR for the risk of dyslipidaemia of 1.58 (95% CI = 1.17, 2.14), compared to those aged <50 years, who had an HR of 1.54 (95% CI = 1.07, 2.20) (Figure S2 in the **Online Supplementary Document**). North America and Europe showed higher risk than Africa (HR = 2.10, 95% CI = 1.67, 2.20; HR = 1.34, 95% CI = 1.08, 1.67) (Figure S3 in the **Online Supplementary Document**). According to follow-up duration, the group after 2015 (HR = 1.51, 95% CI = 1.13, 2.02) showed a greater risk of dyslipidaemia in PLWH (Figure S4 in the **Online Supplementary Document**). Meanwhile, subgroups with a larger proportion of men (>50%) or smokers (≥50%) showed more significant risk of dyslipidaemia (HR = 1.69, 95% CI = 1.41, 2.03; HR = 1.53, 95% CI = 1.15, 2.03) (Figures S5–6 in the **Online Supplementary Document**). We did not observe the correlation between the HR of PLWH having dyslipidaemia and HIV diagnosis and ART duration. While the HR of PLWH having dyslipidaemia showed a significant positive correlation with CD4^+^T cell count (Figure S9 in the **Online Supplementary Document**).

### CAD

The CAD analysis included seven studies involving 9 798 698 patients [28,31,42–46]. The risk of developing CAD was greater in PLWH (HR = 1.37; 95% CI = 1.24, 1.51, *I^2^* = 53.5%) (**Figure 3**), and the prevalence of CAD among PLWH was 24% (**Figure 2**).

The risk of CAD was decreased among the elderly, but there was no discernible difference between groups (≥50 years: HR = 1.28, 95% CI = 1.20, 1.37; <50 years: HR = 1.50, 95% CI = 1.39, 1.62) (Figure S2 in the **Online Supplementary Document**). In comparison to the European group, the North American group displayed a more substantial risk (HR = 1.37; 95% CI = 1.23, 1.53) (Figure S3 in the **Online Supplementary Document**). The risk of developing CAD was lower in the group that was monitored after 2015 (HR = 1.38; 95% CI = 1.23, 1.55) (Figure S4 in **Online Supplementary Document**). The incidence of CAD did not differ between various proportions of smokers (Figure S6 in **Online Supplementary Document**). And the racial-ethnic differences did not affect the risk of CAD (Figure S7 in **Online Supplementary Document**). No correlation was observed between the HR of hypertension in PLWH and ART duration and CD4^+^T cell count (Figure S10 in **Online Supplementary Document**).

### MI

We included 10 studies involving 1 269 005 patients [28,47–53]. MI was more common among PLWH compared to HIV-negative individuals (HR = 1.47; 95% CI = 1.28, 1.68, *I^2^* = 76.8%) (**Figure 3**). The prevalence of MI of 12% was found in PLWH (**Figure 2**). Europe (HR = 1.81; 95% CI = 1.34, 2.44) and North America (HR = 1.35; 95% CI = 1.21, 1.50) had higher risks of MI based on regions (Figure S3 in the **Online Supplementary Document**). MI risk did not vary by follow-up duration (Figure S4 in the **Online Supplementary Document**). All included patients were aged <50 years, hence age subgroups were not analysed. Between the two groups, the proportion of smokers did not significantly differ (Figure S6 in the **Online Supplementary Document**).

**Figure 4:**
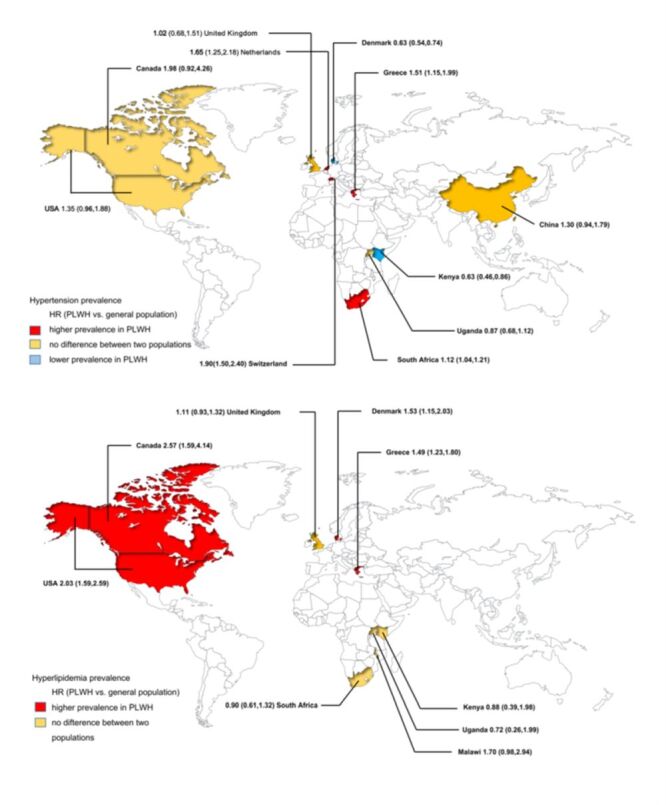
Global distribution of the characteristics of cardiovascular disease risk in PLWH. HR – hazard ratio, PLWH – people living with HIV

To analyse the CVD risk of PLWH globally, we drew a global map to illustrate the disparities in disease risk between regions (**Figure 4**).

### Sensitivity analysis

The results were unaltered by the stepwise exclusion of single studies and pooled analysis of the remaining studies. Hence, the findings of meta-analysis were considered valid (Figure S11 in the **Online Supplementary Document**).

## DISCUSSION

### Principal findings

To the best of our knowledge, this systematic review and meta-analysis comprehensively assessed the prevalence and risk of developing CVD in PLWH. We focused on four typical CVD (hypertension, dyslipidaemia, CAD, and MI) to offer a more comprehensive basis for the clinical monitoring and treatment of CVD in PLWH. Our results were as follows: (1) PLWH had higher risks of hyperlipidaemia, CAD, and MI but not hypertension than the general population. (2) Similar to the general population, hypertension and hyperlipidaemia had a high prevalence among PLWH. (3) Elderly PLWH had a relatively higher risk of hyperlipidaemia, whereas the risk of CAD decreased with age. (4) We observed significant regional differences in the risk of CVD, with North America having the highest CVD risk among PLWH when compared to other countries or regions. (5) The relative risk of CVD decreased with ART initiation after 2015 (i.e. rapid ART, ART advancement). (6) The prevalence of dyslipidaemia was correlated with CD4^+^T cell count.

Similar to evidence from other studies [54], our meta-analysis showed that PLWH had a greater chance of developing dyslipidaemia than the general population. A previous study reported that ART was linked to modest weight gain and elevated total cholesterol and triglyceride levels [55]. However, the mechanisms underlying dyslipidaemia in PLWH remain unclear. Additionally, PLWH had a higher chance of developing CAD, which is similar to previous studies reporting that HIV infection raises the risk of CAD [53,56], but the Swiss HIV Cohort Study found that HIV infection reduced the risk of CAD by 0.5–0.8-fold [57]. These inconsistent results may be attributable to the different study regions and the limited sample size of the Swiss study. Thus, additional studies are required to substantiate the link between CAD risk and HIV infection. Studies showed that the risks of MI or stroke were 1.5–2 times greater in PLWH compared to the general population [58]. Like our findings, PLWH had a 2-fold greater risk of acute MI than the general population in a cohort without traditional CVD risk factors [59]. Surprisingly, PLWH and the general population had similar hypertension risk. Regarding the connection between HIV infection and hypertension, various studies, however, have come to conflicting results. Compared to uninfected controls, the prevalence rates of hypertension in earlier studies were lower [60], comparable [61], and higher [62] in PLWH. This may be because the general population has a stronger white-coat effect. People living with HIV may also have better health habits, regular interactions with health care workers, and are more inclined to be watched for hypertension risk factors. Consequently, further prospective researches are needed to examine the connection between HIV infection and hypertension.

Traditional risk factors linked to an elevated risk of CVD include smoking, sex, and age. Our findings demonstrated that age did not affect the relative risk of CVD in PLWH, indicating that younger patients should not be overlooked. Specifically, age enhanced the relative risk of dyslipidaemia. This may be because, with age, the cardiovascular system can undergo structural changes, arterial elasticity gradually decreases, serum cholesterol levels increase, and coronary artery disease is more likely to occur. Moreover, elderly are more sensitive to medications and experience more serious drug reactions. These changes may aggravate aging society’s personal and social medical burdens. Monitoring public health measures like screening in primary care has been shown to be help achieve the goal of ‘compression of morbidity’ [63]. However, young people are more likely to develop CAD, possibly due to relatively longer working hours and greater financial pressures. Men may have a higher risk of CVD because of social pressures, stigmatization and smoking, all of which can accelerate CVD onset and progression. Special attention to social stress and smoking status is warranted for CVD risk in PLWH.

The burden of HIV-related CVD has risen globally over the past 20 years, with major regional disparities. These disparities are most pronounced in the Asia-Pacific and sub-Saharan Africa regions [64]. Subgroup global analysis revealed that PLWH in North America had a higher CVD risk than those in other countries and regions, including Africa, whereas PLWH in Europe have a higher MI risk. Lifestyle, an underlying risk factor for CVD development, may have played an important role in these results. In North America, owing to differences in dietary habits and lifestyle, food is rich in saturated fats and high in cholesterol, coupled with life stress, sedentary behaviour, and reduced exercise. The risk of CVD was raised by these factors. Second, all diseases (including hepatitis C and depression) are expected to stimulate systemic immune activation and inflammation in women living with HIV in North America and Europe [65]. According to a worldwide study of cardiovascular risk in eight regions [66], the highest prevalence of overweight was found in Latin America, whereas hypertension and high cholesterol were common in Europe. Moreover, Latin America and Eastern Europe were notably affected by the risk factor of smoking. Combining these factors may prevent CVD associated with HIV. While there was no difference in the risk of CVD between racial groups, such as blacks and whites.

Although the literature search was conducted after 2015, some studies included patients treated with ART before 2015 and the application of ART varied widely. Older ART regimens commonly used in low-income areas (e.g. lopinavir, abacavir, and ritonavir) have side effects that compromise the cardiovascular system, including disturbances in lipid metabolism. In contrast, some regimens (e.g. dolutegravir or atazanavir) may have fewer adverse effects on the cardiovascular system [67]. Intriguingly, in the present study, the risk ratio for CVD decreased after 2015. We assume that with the improvement of ART and the promotion of ‘treatment after discovery’ policies, the side effects of ART have gradually decreased. One randomised controlled trial (RCT) reported that boosted atazanavir treatment resulted in a reduced incidence of carotid intima-media thickness progression than darunavir and ritonavir therapy [68]. In addition, the novel pathophysiological mechanisms of CVD in PLWH may involve immune reconstitution, microbial translocation, and chronic inflammation [69,70]. Inflammation and immune cell activation have also been shown to play atherogenic roles in HIV infection [71]. The promotion of ‘treatment after discovery’ can promptly suppress inflammation, effectively inhibit HIV replication, and promote immune system recovery and reconstruction, thus improving the accumulation of lipids in the body, which also supports our hypothesis.

Our analysis revealed a noteworthy observation regarding the impact of HIV infection on the immune system. Specifically, the virus leads to a reduction in CD4^+^T cell counts and compromises immune function. Interestingly, our findings demonstrate a strong positive correlation between dyslipidaemia and CD4^+^T cell counts, aligning with previous research [72]. Elevated CD4^+^T cell counts were linked to higher lipid levels, and naive CD4^+^T cells showed a positive association with peripheral blood hypercholesterolemia. This underscores the significance of immune status changes in health recovery. With the use of ART, obesity was also more common in PLWH, and CD4^+^T cell counts increased with normal weight and obesity [73]. These results emphasised the importance of regularly monitoring CD4^+^T cell counts in PLWH to potentially reduce the risk of CVD. Given the limited number of studies available, future prospective research is warranted to validate these findings.

Traditional risk factors, the direct effects of HIV infection, and the negative side effects of some antiretroviral drugs may contribute to the pathogenesis of CVD in PLWH. Non-traditional CVD risk factors including the development status of the country and an individual's body mass index also increase the risk of CVD. Regardless of HIV infection, these risk variables increase exponentially along with the risk of CVD. The immune system is crucial for development and progression of CVD in PLWH. The bulk of research included in the current analysis were cross-sectional or cohort studies, which limits our understanding of HIV-associated CVD. Higher-level evidence such as that from RCTs is currently lacking. Therefore, additional studies are necessary to confirm these findings.

### Limitations

This study has several limitations. First, although CVD risk ratio was adjusted, residual confounding factors were possible. The main reasons for this lie in the higher frequency of modifiable and non-modifiable cardiovascular risks, as shown in previous studies [74]. Other confounders were equally adjusted in different studies. Second, pooled relative risk estimates were used to calculate CAD risk in PLWH, which represented the overall risk for all types of plaque in the coronary arteries but did not particularly include estimates for carotid plaque, angina, or other chronic atherosclerotic disease symptoms. Nevertheless, angina has a negligible impact on people with ischemic heart disease [75]. Therefore, this limitation had little impact on the estimated total burden. Finally, substantial heterogeneity was observed in the pooled risk ratios. Given that most data are based on global statistics, the cause of this heterogeneity is probably multifaceted and reflects variations in demography, sample size, and case ascertainment bias.

## CONCLUSIONS

People living with HIV had a higher risk of CVD than the general population, particularly in North America and Europe. However, there were no appreciable differences in the risk of hypertension between groups. Additionally, sex, age, and tobacco use influenced the risk of CVD. As PLWH age in the post-ART era, CVD poses additional challenges for clinicians and health departments. Our estimates have important policy implications for facilitating access to health care and are now accepted recommendations for early initiation of ART. Further research is required, especially in resource-limited communities and developing countries with high rates of HIV infection, such as South Africa.

## Additional material


Online Supplementary Document
