# Erratum: San Z, Wenjing W, Jiaze H, Wenshan D, Xiaoran M, Honglin G, Yaxin W, Sibo L, Yanbing L, Tian T, Wenjun K, Dongxia W, Tong Z, Xiaojie H. Higher cardiovascular disease risks in people living with HIV: A systematic review and meta-analysis. J Glob Health 2024;14:04078.

**DOI:** 10.7189/jogh.16.1404078err1

**Published:** 2026-05-22

**Authors:** Journal of Global Health Editorial Team



Following the publication of the manuscript ‘Higher cardiovascular disease risks in people living with HIV: A systematic review and meta-analysis’ on 26 April 2024, the authors noted during institutional archiving and verification of the published article metadata that the order of institutional affiliations and the corresponding author-affiliation superscript numbers did not accurately reflect the institution where the majority of the work was conducted. They also identified two typographical errors in affiliation 6. In addition, in the ‘Cite as’ section, the author names were incorrectly presented in the format ‘Given name Family-name initial’. The correct format is ‘Family name Given name initial’.

Specifically, the affiliation ‘Clinical and Research Center for Infectious Diseases, Beijing Youan Hospital, Capital Medical University, Beijing, PR China’ was listed as affiliation 2 but should have been listed as affiliation 1, while ‘West China School of Medicine, Sichuan University, Chengdu, Sichuan, PR China’ was listed as affiliation 1 but should have been listed as affiliation 2. In addition, typographical errors were identified in affiliation 6: ‘Department of Opthalmology, Beijing Youan Hosptial’. The word ‘Opthalmology’ should read ‘Ophthalmology’ and ‘Hosptial’ should read ‘Hospital’. In the ‘Cite as’ section, the incorrectly presented names were ‘San Z, Wenjing W, Jiaze H, Wenshan D, Xiaoran M, Honglin G, Yaxin W, Sibo L, Yanbing L, Tian T, Wenjun K, Dongxia W, Tong Z, Xiaojie H’. The corrected version reads ‘Zhu S, Wang W, He J, Duan W, Ma X, Guan H, Wu Y, Li S, Li Y, Tian T, Kong W, Wu D, Zhang T, Huang X’.

The affiliation order has been corrected, the author-affiliation superscript numbers have been updated accordingly, and the typographical errors in affiliation 6 and the ‘Cite as’ section have been corrected in both the PDF and online versions. The authors regret these errors, take full responsibility, and apologise for any inconvenience caused to readers or the authors.

These corrections have been made to the PDF and online versions of the manuscript on 22 May 2026, following notification from the authors’ notice on 3 May 2026.

