# Supplementary material for: Higher cardiovascular disease risks in people living with HIV: A systematic review and meta-analysis
Source: J Glob Health. 2024 Apr 26;14:04078. doi: 10.7189/jogh.14.04078 (PMC11046517; doi:10.7189/jogh.14.04078)
Supplement: Online Supplementary Document [file jogh-14-04078-s001.pdf]

# Supplemental Content

**San Zhu[1,2#], BMed, Wenjing Wang[2#], BMed, Jiaze He[2#], MD, Wenshan Duan[2], BMed, Xiaoran Ma[3], Honglin Guan[4], BMed, Yaxin Wu[2], MD, Sibo Li[2], BMed, Yanbing Li[5], PhD, Tian Tian[5], MD, Wenjun Kong[6], PhD, Dongxia Wu[2], MD, Tong Zhang[2], PhD, Xiaojie Huang[2*], MD, PhD**

**Author Affiliations**

1 West China School of Medicine, Sichuan University, Chengdu, Sichuan 610065, PR China

2 Clinical and Research Center for Infectious Diseases, Beijing Youan Hospital, Capital Medical University, Beijing 100069, PR China

3 Tianjin University, Tianjin 300072, PR China

4 Hematology Department, The First Affiliated Hospital of China Medical University, China Medical University, No. 155, Nanjing North Street, Heping District, Shenyang, Liaoning Province 110001, PR China

5 Cardiovascular Department, Beijing Youan Hospital, Capital Medical University, No.8 Xitoutiao, Youanmenwai, Feng Tai District, Beijing 100069, PR China

6 Department of Opthalmology, Beijing Youan Hosptial, Capital Medical University, Beijing, 100069, PR China

*** Corresponding author:**

Email: Xiaojie Huang, huangxiaojie78@ccmu.edu.cn

Postal address: Clinical and Research Center for Infectious Diseases, Beijing Youan Hospital, Capital Medical University, No.8 Xitoutiao, Youanmenwai, Feng Tai District, Beijing 100069, PR China

# S Zhu, WJ Wang, and JZ He contributed equally.


| | |
|---|---|
| **Table S1.** The search strategy in PubMed, Web of Science, Cochrane Library, Embase | P2 |
| **Table S2**. Detailed search strategies (between January 1, 2015, and May 12,2023) | P6 |
| **Table S3.** ICD-10 and ICD-11 Code Used for Screening for Chronic Medical Condition Diagnoses | P12 |
| **Table S4.** Standardized definitions of diagnoses | P13 |
| **Table S5.** Inclusion and Exclusion Criteria of studies | P14 |
| **Table S6.** Risk of bias assessment | P16 |
| **eFigure 1.** Funnel plots | P19 |
| **eFigure2.** Subgroup analysis based on different age | P20 |
| **eFigure3.** Subgroup analysis based on different region | P21 |
| **eFigure4.** Subgroup analysis based on different follow-up | P22 |
| **eFigure5.** Subgroup analysis based on the proportion of male | P23 |
| **eFigure6.** Subgroup analysis based on different smoking | P23 |
| **eFigure7.** Sensitivity analysis | P24 |


**This supplemental material has been provided by the authors to give readers additional information about their work.**

## Table S1. The search strategy in PubMed, Web of Science, Cochrane Library, Embase

| PUBMED |
| --- |
| CORONARY HEART DISEASE STUDIES:<br>((Coronary heart disease?[Title/Abstract] OR Atherosclerosis [MeSH Terms] OR Coronary artery disease? [Title/Abstract]) AND (Antiretroviral therapy [Title/Abstract] OR ART [Title/Abstract] OR Antiretroviral therapy,highly active [MeSH Terms] OR HIV [Title/Abstract] OR Human immunodeficiency virus [MeSH Terms]) AND ((RR[Title/Abstract]) OR (OR) OR (HR[Title/Abstract]) OR (odds ratio[Title/Abstract]) OR (relative risk[Title/Abstract]) OR (risk ratio[Title/Abstract]) OR (hazard ratio[Title/Abstract]))) |
| ACUTE MYOCARDIAL INFARCTION (AMI) STUDIES<br>(Myocardial Infarct* [Title/Abstract] OR Heart Infarct* [Title/Abstract] OR AMI [Title/Abstract] OR Myocardial Infarction [MeSH Terms] OR Coronary Thrombosis [MeSH Terms]) AND (Antiretroviral therapy [Title/Abstract] OR ART [Title/Abstract] OR Antiretroviral therapy, highly active [MeSH Terms] OR HIV [Title/Abstract] OR Human immunodeficiency virus [MeSH Terms]) AND ((RR[Title/Abstract]) OR (OR) OR (HR[Title/Abstract]) OR (odds ratio[Title/Abstract]) OR (relative risk[Title/Abstract]) OR (risk ratio[Title/Abstract]) OR (hazard ratio[Title/Abstract]))) |
| HYPERLIPIDEMIAS STUDIES<br>(Hypercholesterolemia [MeSH Terms] OR Dyslipidemias [MeSH Terms] OR hyperlipidaemia [Title/Abstract] OR Hypercholesteremia [Title/Abstract] OR elevated cholesterol [Title/Abstract]) AND (Antiretroviral therapy [Title/Abstract] OR ART [Title/Abstract] OR Antiretroviral therapy, highly active [MeSH Terms] OR HIV [Title/Abstract] OR Human immunodeficiency virus [MeSH Terms]) AND ((RR[Title/Abstract]) OR (OR) OR (HR[Title/Abstract]) OR (odds ratio[Title/Abstract]) OR (relative risk[Title/Abstract]) OR (risk ratio[Title/Abstract]) OR (hazard ratio[Title/Abstract]))) |
| HYPERTENSION STUDIES<br>(Hypertension [MeSH Terms] OR high blood pressure [Title/Abstract] OR systolic hypertension [Title/Abstract] OR diastolic hypertension [Title/Abstract] OR anti-hypertensive [Title/Abstract] OR hypertensive [Title/Abstract]) AND (Antiretroviral therapy [Title/Abstract] OR ART [Title/Abstract] OR Antiretroviral therapy, highly active [MeSH Terms] OR HIV [Title/Abstract] OR Human immunodeficiency virus [MeSH Terms]) AND ((RR[Title/Abstract]) OR (OR) OR (HR[Title/Abstract]) OR (odds ratio[Title/Abstract]) OR (relative risk[Title/Abstract]) OR (risk ratio[Title/Abstract]) OR (hazard ratio[Title/Abstract]))) |

| Web of Science |
| --- |
| CORONARY HEART DISEASE STUDIES<br>#1 TS=((Coronary heart disease) OR (Atherosclerosis) OR (Coronary artery disease))<br>#2 TS=("Antiretroviral therapy" OR ART OR HIV)<br>#3 TS=(RR OR (OR) OR HR OR (odds ratio) OR (relative risk) OR (risk ratio) OR (hazard ratio))<br>#4 #1 AND #2 AND #3 |

| |
|---|
| ACUTE MYOCARDIAL INFARCTION (AMI) STUDIES<br>#5 TS=((Myocardial Infarct*) OR (Heart Infarct*) OR AMI OR “Myocardial Infarction” OR “Coronary Thrombosis”)<br>#6 #5 AND #2 AND #3 |
| HYPERLIPIDEMIAS STUDIES<br>#7 TS=(Hypercholesterolemia OR Dyslipidemias OR hyperlipidaemia OR Hypercholesteremia OR “elevated cholesterol”)<br>#8 #7 AND #2 AND #3 |
| HYPERTENSION STUDIES<br>#9 TS=(Hypertension OR “high blood pressure” OR “systolic hypertension” OR “diastolic hypertension” OR “anti-hypertensive” OR hypertensive)<br>#10 #9 AND #2 AND #3 |

| **Cochrane Library** |
|---|
| CORONARY HEART DISEASE STUDIES<br>#1 (“Coronary heart disease”):ti,ab,kw<br>#2 MeSH descriptor: [Atherosclerosis]<br>#3 (“Coronary artery disease”):ti,ab,kw<br>#4 (Antiretroviral therapy):ti,ab,kw<br>#5 (ART):ti,ab,kw<br>#6 (HIV):ti,ab,kw<br>#7 MeSH descriptor: [Antiretroviral Therapy, Highly Active]<br>#8 MeSH descriptor: [HIV] explode all trees<br>#9 (RR):ti,ab,kw<br>#10 (HR):ti,ab,kw<br>#11 (odds ratio):ti,ab,kw<br>#12 (relative risk):ti,ab,kw<br>#13 (risk ratio):ti,ab,kw<br>#14 (hazard ratio):ti,ab,kw<br>#15 #1 OR #2 OR #3<br>#16 #4 OR #5 OR #6 OR #7 OR #8<br>#17 #9 OR #10 OR #11 OR #12 OR #13 OR #14<br>#18 #15 AND #16 AND #17 |
| ACUTE MYOCARDIAL INFARCTION (AMI) STUDIES<br>#19 (“Myocardial Infarction”):ti,ab,kw<br>#20 (“Heart Infarction”):ti,ab,kw<br>#21 (AMI):ti,ab,kw<br>#22 MeSH descriptor: [Coronary Thrombosis]<br>#23 MeSH descriptor: [Myocardial Infarction]<br>#24 #19 OR #20 OR #21 OR #22 OR #23<br>#25 #24 AND #16 AND #17 |
| HYPERLIPIDEMIAS STUDIES<br>#26 (hyperlipidaemia):ti,ab,kw<br>#27 MeSH descriptor: [Hypercholesterolemia] |

| |
|---|
| #28 (Hypercholesteremia):ti,ab,kw<br>#29 ("elevated cholesterol"):ti,ab,kw<br>#30 MeSH descriptor: [Dyslipidemias]<br>#31 #26 OR #27 OR #28 OR #29 OR #30<br>#32 #31 AND #16 AND #17 |
| HYPERTENSION STUDIES<br>#33 MeSH descriptor: [Hypertension]<br>#34 ("high blood pressure"):ti,ab,kw<br>#35 ("systolic hypertension"):ti,ab,kw<br>#36 ("diastolic hypertension"):ti,ab,kw<br>#37 (anti-hypertensive):ti,ab,kw<br>#38 (hypertensive):ti,ab,kw<br>#39 #33 OR #34 OR #35 OR #36 OR #37 OR #38<br>#40 #39 AND #16 AND #17 |

| **Embase** |
|---|
| CORONARY HEART DISEASE STUDIES<br>#1 'coronary heart disease':ab,ti,kw<br>#2 'atherosclerosis'/exp<br>#3 'coronary artery atherosclerosis'/exp<br>#4 'coronary artery disease':ab,ti,kw<br>#5 'antiretroviral therapy':ti,ab,kw<br>#6 art:ti,ab,kw<br>#7 hiv:ti,ab,kw<br>#8 'highly active antiretroviral therapy'/exp<br>#9 'human immunodeficiency virus infection'/exp<br>#10 RR:ti,ab,kw<br>#11 HR:ti,ab,kw<br>#12 'odds ratio':ti,ab,kw<br>#13 'relative risk':ti,ab,kw<br>#14 'risk ratio':ti,ab,kw<br>#15 'hazard ratio':ti,ab,kw<br>#16 #1 OR #2 OR #3 OR #4<br>#17 #5 OR #6 OR #7 OR #8 OR #9<br>#18 #10 OR #11 OR #12 OR #13 OR #14 OR #15<br>#19 #16 AND #17 AND #18 |
| ACUTE MYOCARDIAL INFARCTION (AMI) STUDIES<br>#20 'myocardial Infarction':ti,ab,kw<br>#21 'heart Infarction':ti,ab,kw<br>#22 ami:ti,ab,kw<br>#23 'heart infarction'/exp<br>#24 'coronary artery thrombosis'/exp<br>#25 #20 OR #21 OR #22 OR #23 OR #24<br>#26 #25 AND #17 AND #18 |

| |
| --- |
| HYPERLIPIDEMIAS STUDIES<br>#27 hyperlipidaemia:ti,ab,kw<br>#28 ‘hyperlipidemia’/exp<br>#29 ‘elevated cholesterol’:ti,ab,kw<br>#30 ‘dyslipidemia'/exp<br>#31 #27 OR #28 OR #29 OR #30<br>#32 #31 AND #17 AND #18 |
| HYPERTENSION STUDIES<br>#33 ‘hypertension'/exp<br>#34 ‘high blood pressure':ti,ab,kw<br>#35 ‘systolic hypertension':ti,ab,kw<br>#36 ‘diastolic hypertension':ti,ab,kw<br>#37 anti-hypertensive:ti,ab,kw<br>#38 hypertensive:ti,ab,kw<br>#39 #33 OR #34 OR #35 OR #36 OR #37 OR #38<br>#40 #39 AND #17 AND #18 |

**Table S2. Detailed search strategies (between January 1, 2015, and May 12,2023)**

| **PubMed (1587)** | | | **Web of Science (1368)** | | | **Cochrane Library (150)** | | | **Embase (2476)** | | |
|---|---|---|---|---|---|---|---|---|---|---|---|
| Coronary Heart Disease Studies | | | | | | | | | | | |
| #1 | Coronary heart disease?[Title/Abstract] | 14620 | #1 | TS=((Coronary heart disease) OR (Atherosclerosis) OR (Coronary artery disease)) | 166338 | #1 | ("Coronary heart disease"):ti,ab,kw | 4151 | #1 | coronary heart disease':ab,ti,kw | 24020 |
| #2 | Atherosclerosis [MeSH Terms] | 27924 | #2 | TS=("Antiretroviral therapy" OR ART OR HIV) | 473951 | #2 | MeSH descriptor: [Atherosclerosis] | 3439 | #2 | atherosclerosis'/exp | 124982 |
| #3 | Coronary artery disease? [Title/Abstract] | 39429 | #3 | TS=(RR OR HR OR (odds ratio) OR (relative risk) OR (risk ratio) OR (hazard ratio)) | 635296 | #3 | (“Coronary artery disease”):ti,ab,kw | 13034 | #3 | coronary artery atherosclerosis'/exp | 15758 |
| #4 | Antiretroviral therapy [Title/Abstract] | 22806 | #4 | #1 AND #2 AND #3 | 392 | #4 | (Antiretroviral therapy):ti,ab,kw | 5744 | #4 | coronary artery disease':ab,ti,kw | 73196 |
| #5 | ART [Title/Abstract] | 94603 | | | | #5 | (ART):ti,ab,kw | 8566 | #5 | antiretroviral therapy':ti,ab,kw | 32213 |
| #7 | HIV [Title/Abstract] | 115708 | | | | #7 | MeSH descriptor: [Antiretroviral Therapy, Highly | 542 | #7 | hiv:ti,ab,kw | 179120 |

| | | | | | | | | | | | |
|---|---|---|---|---|---|---|---|---|---|---|---|
| | | | | | | | Active] | | | | |
| #8 | Human immunodeficiency virus [MeSH Terms] | 23238 | | | | #8 | MeSH descriptor: [HIV] explode all trees | 1246 | #8 | highly active antiretroviral therapy'/exp | 8832 |
| #9 | RR[Title/Abstract] | 53250 | | | | #9 | (RR):ti,ab,kw | 13322 | #9 | human immunodeficiency virus infection'/exp | 230260 |
| #10 | OR[Title/Abstract] | 3837739 | | | | #10 | (HR):ti,ab,kw | 39657 | #10 | RR:ti,ab,kw | 83594 |
| #11 | HR[Title/Abstract] | 149832 | | | | #11 | (odds ratio):ti,ab,kw | 17870 | #11 | HR:ti,ab,kw | 282945 |
| #12 | odds ratio[Title/Abstract] | 168984 | | | | #12 | (relative risk):ti,ab,kw | 16480 | #12 | odds ratio':ti,ab,kw | 217607 |
| #13 | relative risk[Title/Abstract] | 28619 | | | | #13 | (risk ratio):ti,ab,kw | 34812 | #13 | relative risk':ti,ab,kw | 41212 |
| #14 | risk ratio[Title/Abstract] | 17500 | | | | #14 | (hazard ratio):ti,ab,kw | 20192 | #14 | risk ratio':ti,ab,kw | 22815 |
| #15 | hazard ratio[Title/Abstract] | 91824 | | | | #15 | #1 OR #2 OR #3 | 19230 | #15 | hazard ratio':ti,ab,kw | 129065 |
| #16 | #1 OR #2 OR #3 | 78290 | | | | #16 | #4 OR #5 OR #6 OR #7 OR #8 | 25170 | #16 | #1 OR #2 OR #3 OR #4 | 204761 |
| #17 | #4 OR #5 OR #6 OR #7 OR #8 | 196548 | | | | #17 | #9 OR #10 OR #11 OR #12 OR #13 OR #14 | 99042 | #17 | #5 OR #6 OR #7 OR #8 OR #9 | 399136 |
| #18 | #9 OR #10 OR #11 OR #12 OR #13 OR | 3989732 | | | | #18 | #15 AND #16 AND #17 | 30 | #18 | #10 OR #11 OR #12 OR #13 OR #14 OR | 653195 |

| | | | | | | | | | | | |
|---|---|---|---|---|---|---|---|---|---|---|---|
| | #14 OR #15 | | | | | | | | | #15 | |
| #19 | #16 AND #17 AND #18 | 440 | | | | | | | #19 | #16 AND #17 AND #18 | 307 |
| Acute Myocardial Infarction (AMI) Studies | | | | | | | | | | | |
| #20 | Myocardial Infarct* [Title/Abstract] | 73635 | #5 | TS=((Myocardial Infarct*) OR (Heart Infarct*) OR AMI OR "Myocardial Infarction" OR "Coronary Thrombosis") | 1215525 | #19 | ("Myocardial Infarction"):ti,ab,kw | 19726 | #20 | myocardial Infarction':ti,ab,kw | 127442 |
| #21 | Heart Infarct* [Title/Abstract] | 67 | #6 | #5 AND #2 AND #3 | 341 | #20 | ("Heart Infarction"):ti,ab,kw | 11118 | #21 | heart Infarction':ti,ab,kw | 69 |
| #22 | AMI [Title/Abstract] | 8842 | | | | #21 | (AMI):ti,ab,kw | 1772 | #22 | ami:ti,ab,kw | 16227 |
| #23 | Myocardial Infarction [MeSH Terms] | 42552 | | | | #22 | MeSH descriptor: [Coronary Thrombosis] | 268 | #23 | heart infarction'/exp | 175968 |
| #24 | Coronary Thrombosis [MeSH Terms] | 1687 | | | | #23 | MeSH descriptor: [Myocardial Infarction] | 5741 | #24 | coronary artery thrombosis'/exp | 2121 |
| #25 | #20 OR #21 OR #22 OR #23 OR #24 | 84043 | | | | #24 | #19 OR #20 OR #21 OR #22 OR #23 | 22762 | #25 | #20 OR #21 OR #22 OR #23 OR #24 | 195412 |
| # | #25 AND | 361 | | | | # | #24 AND | 63 | # | #25 AND | 39 |

| | | | | | | | | | | | |
|---|---|---|---|---|---|---|---|---|---|---|---|
| 26 | #17 AND #18 | | | | | 25 | #16 AND #17 | | 26 | #17 AND #18 | 9 |
| Hyperlipidemia Studies | | | | | | | | | | | |
| #27 | Hypercholesterolemia [MeSH Terms] | 4328 | #7 | TS=(Hypercholesterolemia OR Dyslipidemias OR hyperlipidaemia OR Hypercholesteremia OR "elevated cholesterol") | 16822 | #26 | (hyperlipidaemia):ti,ab,kw | 3337 | #27 | hyperlipidaemia:ti,ab,kw | 2708 |
| #28 | Dyslipidemias [MeSH Terms] | 19750 | #8 | #7 AND #2 AND #3 | 37 | #27 | MeSH descriptor: [Hypercholesterolemia] | 1763 | #28 | hyperlipidemia'/exp | 78870 |
| #29 | hyperlipidaemia [Title/Abstract] | 1410 | | | | #28 | (Hypercholesteremia):ti, ab,kw | 39 | #29 | elevated cholesterol':ti,ab,kw | 612 |
| #30 | Hypercholesteremia [Title/Abstract] | 125 | | | | #29 | ("elevated cholesterol") :ti,ab,kw | 109 | #30 | dyslipidemia'/exp | 57730 |
| #31 | elevated cholesterol [Title/Abstract] | 334 | | | | #30 | MeSH descriptor: [Dyslipidemias] | 4189 | #31 | #27 OR #28 OR #29 OR #30 | 131387 |
| #32 | #27 OR #28 OR #29 OR #30 OR #31 | 21075 | | | | #31 | #26 OR #27 OR #28 OR #29 OR #30 | 7052 | #32 | #31 AND #17 AND #18 | 415 |
| #33 | #32 AND #17 AND #18 | 207 | | | | #32 | #31 AND #16 AND #17 | 18 | | | |
| Hypertension Studies | | | | | | | | | | | |

| | | | | | | | | | | | |
|---|---|---|---|---|---|---|---|---|---|---|---|
| #34 | Hypertension [MeSH Terms] | 69226 | #9 | TS=(Hypertension OR "high blood pressure" OR "systolic hypertension" OR "diastolic hypertension" OR "anti-hypertensive" OR hypertensive) | 228152 | #33 | MeSH descriptor: [Hypertension] | 11072 | #33 | hypertension'/exp | 438855 |
| #35 | high blood pressure [Title/Abstract] | 6678 | #10 | #9 AND #2 AND #3 | 598 | #34 | ("high blood pressure"):ti,ab,kw | 2240 | #34 | high blood pressure': ti,ab,kw | 12134 |
| #36 | systolic hypertension [Title/Abstract] | 578 | | | | #35 | ("systolic hypertension"):ti,ab,kw | 376 | #35 | systolic hypertension':ti,ab,kw | 1088 |
| #37 | diastolic hypertension [Title/Abstract] | 314 | | | | #36 | ("diastolic hypertension"):ti,ab,kw | 51 | #36 | diastolic hypertension':ti,ab,kw | 590 |
| #38 | anti-hypertensive [Title/Abstract] | 2177 | | | | #37 | (anti-hypertensive):ti,ab,kw | 936 | #37 | anti-hypertensive:ti,ab,kw | 4939 |
| #39 | hypertensive [Title/Abstract] | 31769 | | | | #38 | (hypertensive):ti,ab,kw | 8550 | #38 | hypertensive:ti,ab,kw | 63481 |
| #40 | #34 OR #35 OR #36 OR | 87995 | | | | #39 | #33 OR #34 OR #35 OR #36 OR #37 | 18595 | #39 | #33 OR #34 OR #35 OR | 458755 |

| | | | | | | | | | | | |
|---|---|---|---|---|---|---|---|---|---|---|---|
| | #37 OR #38 OR #39 | | | | | | OR #38 | | | #36 OR #37 OR #38 | |
| #41 | #40 AND #17 AND #18 | 579 | | | | #40 | #39 AND #16 AND #17 | 39 | #40 | #39 AND #17 AND #18 | 1355 |

## Table S3. ICD-10 and ICD-11 Code Used for Screening for Chronic Medical Condition Diagnoses

| **Diagnosis** | **ICD-10 Codes** | **ICD-11 Codes** |
|---|---|---|
| Hypertension | I19, I12-I13, I15-I16 | BA00.1，BA00.2，BA00.Y，BA00.Z |
| Dyslipidemia | E78 | 5C80.00，5C80.1，5C80.Z |
| Myocardial infarction | I20-I25, I30-I52, I60-I69, I63 | BA41.0，BA41.1，BA41.Z，BA42.Z |
| Coronary atherosclerosis disease | I25.1 A | BA80.0，BA80.Z |

Abbreviations: ICD, international classification of diseases.

## Table S4. Standardized definitions of diagnoses

| **Chronic Medical Condition** | **Definition** |
|---|---|
| Hypertension | Hypertension was confirmed by use of an antihypertensive medication or >3 blood pressure readings with systolic blood pressure >140 mmHg and/or diastolic blood pressure >90mmHg |
| Dyslipidemia | Hyperlipidemia was confirmed as a total cholesterol >200 mg/dl or prescription of a lipid-lowering medication<br>Use of a lipid-lowering medication, or<br>Any of the following laboratory abnormalities: low-density lipoprotein≥130 mg/dL; high-density lipoprotein≤30 mg/dL for men or≤40 mg/dL for women, or<br>Total cholesterol≥200 mg/dL, or<br>Triglycerides≥150 mg/dL |
| Myocardial infarction | The term acute myocardial infarction should be used when there is acute myocardial injury with clinical evidence of acute myocardial ischaemia and with detection of a rise and/or fall of cTn values with at least one value above the 99th percentile URL and at least one of the following:<br>1. Symptoms of myocardial ischaemia;<br>2. New ischaemic ECG changes;<br>3. Development of pathological Q waves;<br>4. Imaging evidence of new loss of viable myocardium or new regional wall motion abnormality in a pattern consistent with an ischaemic aetiology; |
| Coronary atherosclerosis disease | A chronic inflammatory disease of coronary that causes isch-emic heart disease, which is confirmed by any kind of coronary plaque on CCTA. |

Abbreviations: ECG, electrocardiogram; CCTA, coronary CT angiography.

## Table S5. Inclusion and Exclusion Criteria of studies

| Inclusion | Exclusion |
|---|---|
| **Article Type** | |
| 1. observational studies and RCTs<br>2. published in English | 1. unpublished reports and conference abstracts |
| **Context** | |
| 1. studies about HIV treatment and disease management<br>2. studies presenting the OR, HR, or RR of CVDs (MI, CAD, hypertension, or dyslipidemia) in PLWH and the general population<br>3. results were stratified according to region, age, HIV status, CD4 cell count, pVL levels, ART use, or exposure to a particular ART class or regimen | 1. studies with unadjusted estimates and intermediate, surrogate, or CVD biomarker outcomes<br>2. studies involving animals, children, pregnancy |
| **Definition** | |
| 1. studies using standardized definitions for the diseases presented in eTable 3 and 4 | 1. studies examining non-systemic hypertension (e.g., intracranial, pulmonary, and portal hypertension) or those<br>2. studies recruiting or rejecting patients with conditions associated with hypertension |

Abbreviations: RCT, randomized controlled trial; OR, odds ratio; HR, hazard ratio; RR, risk ratio; CVD, cardiovascular disease; MI, myocardial infarction; CAD, coronary atherosclerosis disease; PLWH, people living with HIV; pVL, plasma viral load; ART, antiretroviral therapy.

**Table S6. Quality assessment of included studies.**

| NEWCASTLE - OTTAWA QUALITY ASSESSMENT SCALE FOR CROSS-SECTIONAL STUDIES | | | | | | | | | |
|---|---|---|---|---|---|---|---|---|---|
| Study | SELECTION | | | | COMPARABILITY | OUTCOME | | Score | Evidence quality |
| | Representativeness of the sample | Sample size | Non-respondents | Ascertainment of the exposure (risk factor) | The subjects in different outcome groups are comparable, based on the study design or analysis. Confounding factors are controlled. Maximum : ☆☆ | Assessment of outcome. Maximum : ☆☆ | Statistical test | | |
| Chow, D et al 2015 | ☆ | ☆ | ☆ | - | ☆ | ☆ | ☆ | 6 | Medium risk of bias |
| Miller, PE et al 2015 | ☆ | ☆ | ☆ | ☆ | ☆ | ☆☆ | ☆ | 8 | Low risk of bias |
| Tripathi, A et al 2015 | ☆ | ☆ | ☆ | ☆ | ☆ | ☆☆ | ☆ | 8 | Low risk of bias |
| van Zoest, RA et al 2016 | ☆ | ☆ | ☆ | ☆ | ☆ | ☆☆ | ☆ | 8 | Low risk of bias |
| Gelpi, M et al 2018 | ☆ | ☆ | ☆ | ☆ | ☆ | ☆☆ | ☆ | 8 | Low risk of bias |
| Mayer, KH et al 2018 | ☆ | ☆ | ☆ | ☆ | ☆ | ☆☆ | ☆ | 8 | Low risk of bias |
| Rücker, SCM et al 2018 | ☆ | ☆ | ☆ | - | ☆ | ☆☆ | ☆ | 7 | Low risk of bias |

| | | | | | | | | | |
|---|---|---|---|---|---|---|---|---|---|
| Manne-Goehler, J et al 2019 | ☆ | ☆ | ☆ | - | ☆ | ☆☆ | ☆ | 7 | Low risk of bias |
| Yang, HY et al 2019 | ☆ | ☆ | ☆ | - | ☆ | ☆ | ☆ | 6 | Medium risk of bias |
| Masyuko, SJ et al 2020 | ☆ | ☆ | ☆ | - | ☆ | ☆☆ | ☆ | 7 | Low risk of bias |
| Touloumi, G et al 2020 | ☆ | ☆ | ☆ | - | ☆ | ☆☆ | ☆ | 7 | Low risk of bias |
| Tilahun, H et al 2021 | ☆ | ☆ | ☆ | - | ☆ | ☆☆ | ☆ | 7 | Low risk of bias |
| Xu, X et al 2021 | ☆ | ☆ | ☆ | ☆ | ☆ | ☆☆ | ☆ | 8 | Low risk of bias |
| Enriquez, R et al2022 | ☆ | ☆ | ☆ | ☆ | ☆ | ☆☆ | ☆ | 8 | Low risk of bias |
| Jones, BI et al 2022 | ☆ | ☆ | ☆ | - | ☆ | ☆☆ | ☆ | 7 | Low risk of bias |
| Mogaka, JN et al 2022 | ☆ | ☆ | ☆ | - | ☆ | ☆☆ | ☆ | 7 | Low risk of bias |
| Morales, DR et al 2022 | ☆ | ☆ | ☆ | ☆ | ☆ | ☆☆ | ☆ | 8 | Low risk of bias |

| NEWCASTLE - OTTAWA QUALITY ASSESSMENT SCALE FOR COHORT STUDIES | | | | | | | | | | |
|---|---|---|---|---|---|---|---|---|---|---|
| **STUDY** | **SELECTION** | | | | **COMPARABILITY** | **OUTCOME** | | | **SCORE** | **Evidence quality** |
| | **Representativeness of the exposed cohort** | **Selection of the non-exposed cohort** | **Ascertainment of exposure** | **Demonstration that outcome of interest was not present at start of study** | **Comparability of Cohorts on the Basis of the Design or Analysis Maximum : ☆☆** | **Assessment of outcome** | **Was follow-up long enough for outcomes to occur** | **Adequacy of follow up of cohorts** | | |
| Badejo, OA et al 2015 | ☆ | ☆ | ☆ | ☆ | ☆ | ☆ | - | ☆ | 7 | Low risk of bias |
| Hasse, B et al 2015 | ☆ | ☆ | ☆ | ☆ | ☆ | ☆ | ☆ | ☆ | 8 | Low risk of bias |
| Kingsley, LA et al 2015 | ☆ | ☆ | ☆ | ☆ | ☆ | ☆ | ☆ | - | 7 | Low risk of bias |
| Klein, DB et al 2015 | ☆ | ☆ | ☆ | ☆ | ☆ | ☆ | ☆ | ☆ | 8 | Low risk of bias |
| Paisible, AL et al 2015 | ☆ | ☆ | ☆ | ☆ | ☆ | ☆ | - | ☆ | 7 | Low risk of bias |
| Rasmussen, LD et al 2015 | ☆ | ☆ | ☆ | ☆ | ☆ | ☆ | ☆ | ☆ | 8 | Low risk of bias |
| Friedman, EE et al 2016 | ☆ | ☆ | ☆ | - | ☆ | ☆ | ☆ | ☆ | 7 | Low risk of bias |

| | | | | | | | | | | |
|---|---|---|---|---|---|---|---|---|---|---|
| Drozd, DR et al 2017 | ☆ | ☆ | ☆ | ☆ | ☆ | ☆ | - | ☆ | 7 | Low risk of bias |
| Alonso, A et al 2019 | ☆ | ☆ | ☆ | ☆ | ☆ | ☆ | ☆ | ☆ | 8 | Low risk of bias |
| Masiá, M et al 2019 | ☆ | ☆ | ☆ | ☆ | ☆ | ☆ | - | ☆ | 7 | Low risk of bias |
| Rosenson, RS et al 2020 | ☆ | ☆ | ☆ | ☆ | ☆ | ☆ | - | ☆ | 7 | Low risk of bias |
| Russell, EAB et al 2020 | ☆ | ☆ | ☆ | ☆ | ☆ | ☆ | ☆ | ☆ | 8 | Low risk of bias |
| Tarr, PE et al 2020 | ☆ | ☆ | ☆ | ☆ | ☆ | ☆ | ☆ | ☆ | 8 | Low risk of bias |
| Gooden, TE et al 2022 | ☆ | ☆ | ☆ | ☆ | ☆ | ☆ | ☆ | ☆ | 8 | Low risk of bias |

## Figure S1. Funnel plots.

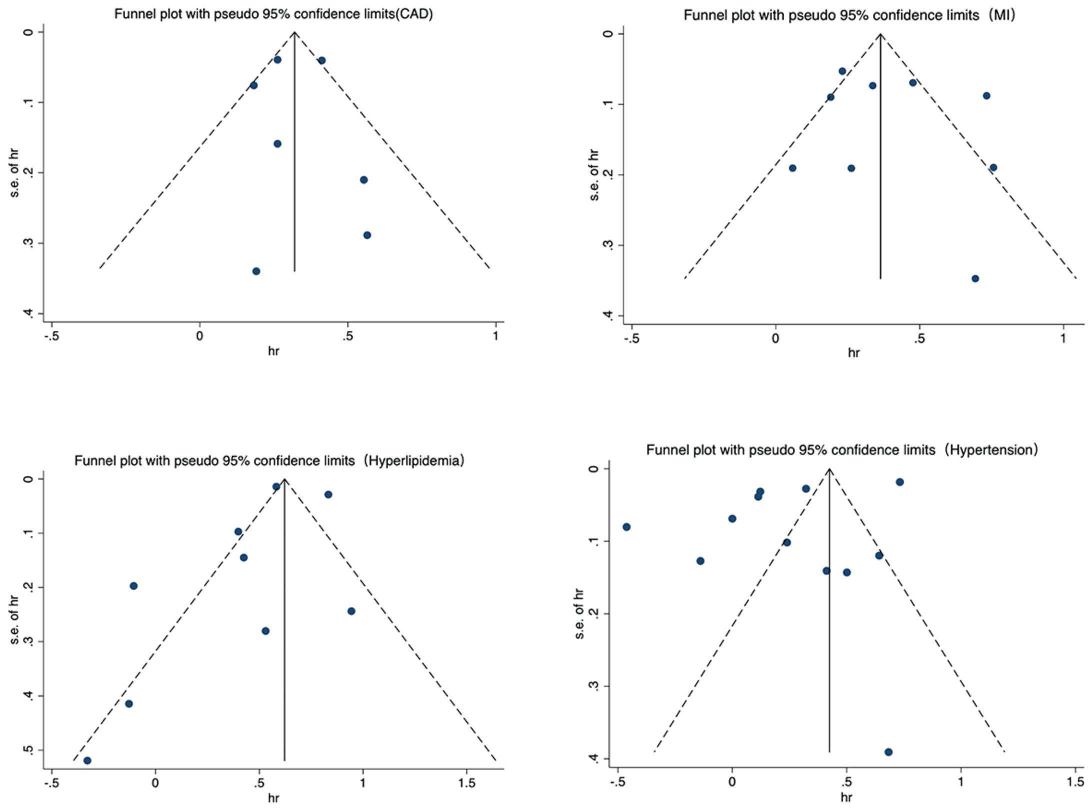


Abbreviations: CAD, coronary atherosclerosis disease; MI, myocardial infarction.

(A) Hypertension; (B) Hyperlipidemia; (C) CAD; (D) MI;

# Figure S2. Subgroup analysis based on different age.

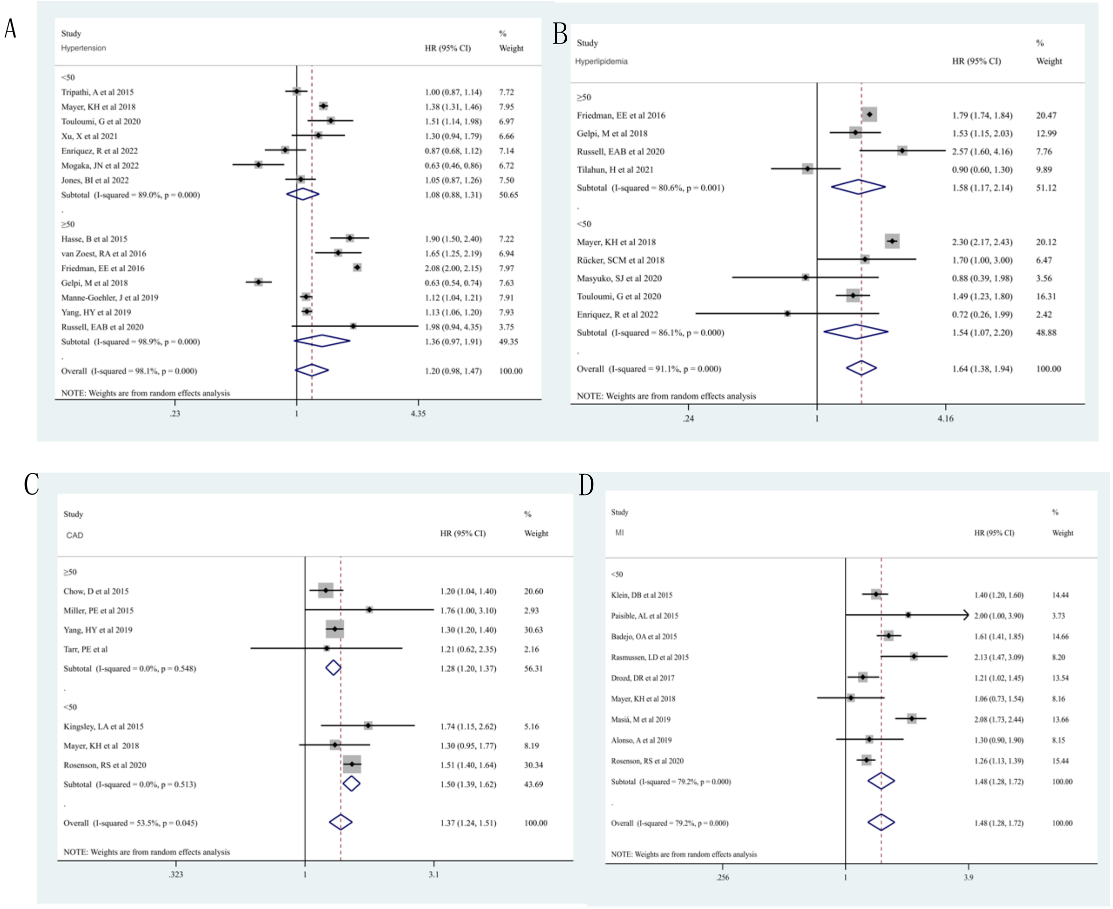


Abbreviations: CAD, coronary atherosclerosis disease; MI, myocardial infarction.

(A) Hypertension; (B) Hyperlipidemia; (C) CAD; (D) MI;

## Figure S3. Subgroup analysis based on different region.

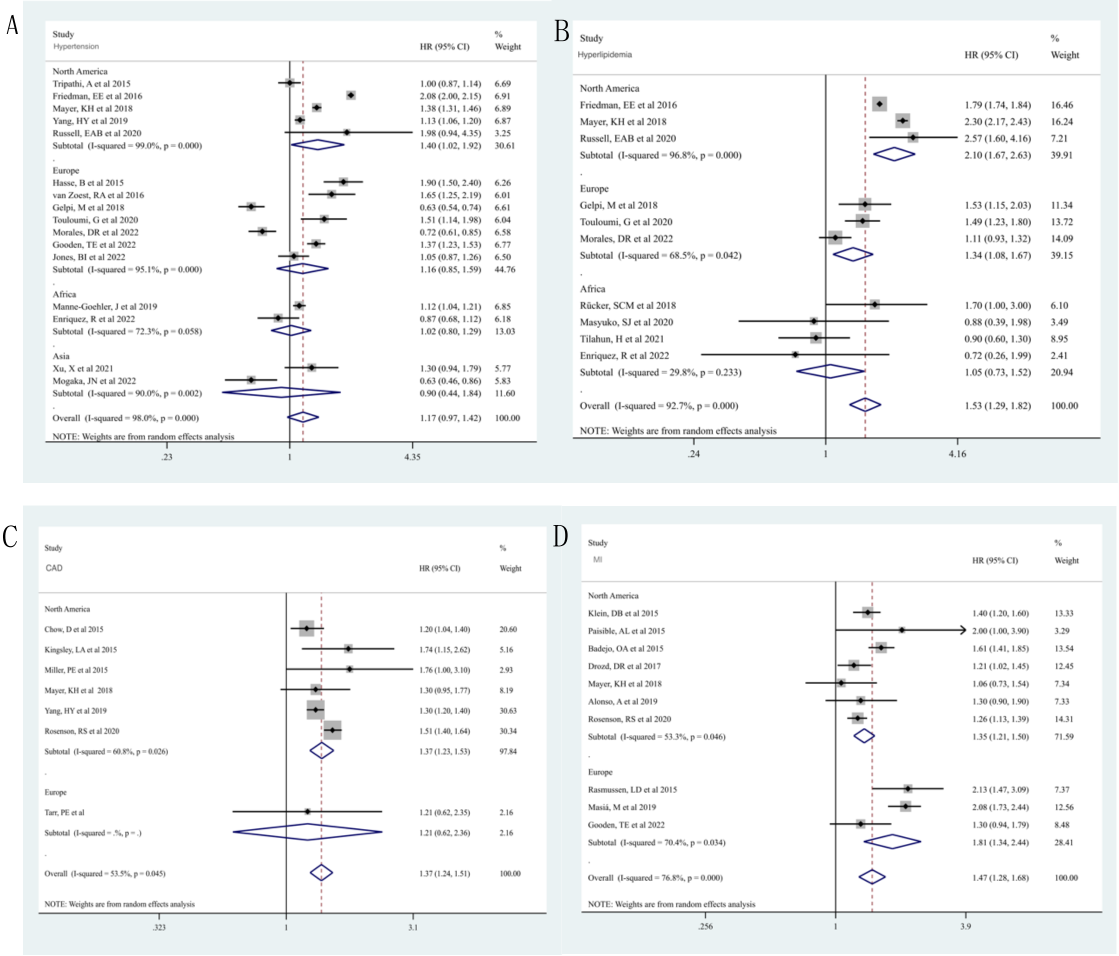


Abbreviations: CAD, coronary atherosclerosis disease; MI, myocardial infarction.

(A) Hypertension; (B) Hyperlipidemia; (C) CAD; (D) MI;

## Figure S4. Subgroup analysis based on different follow-up.

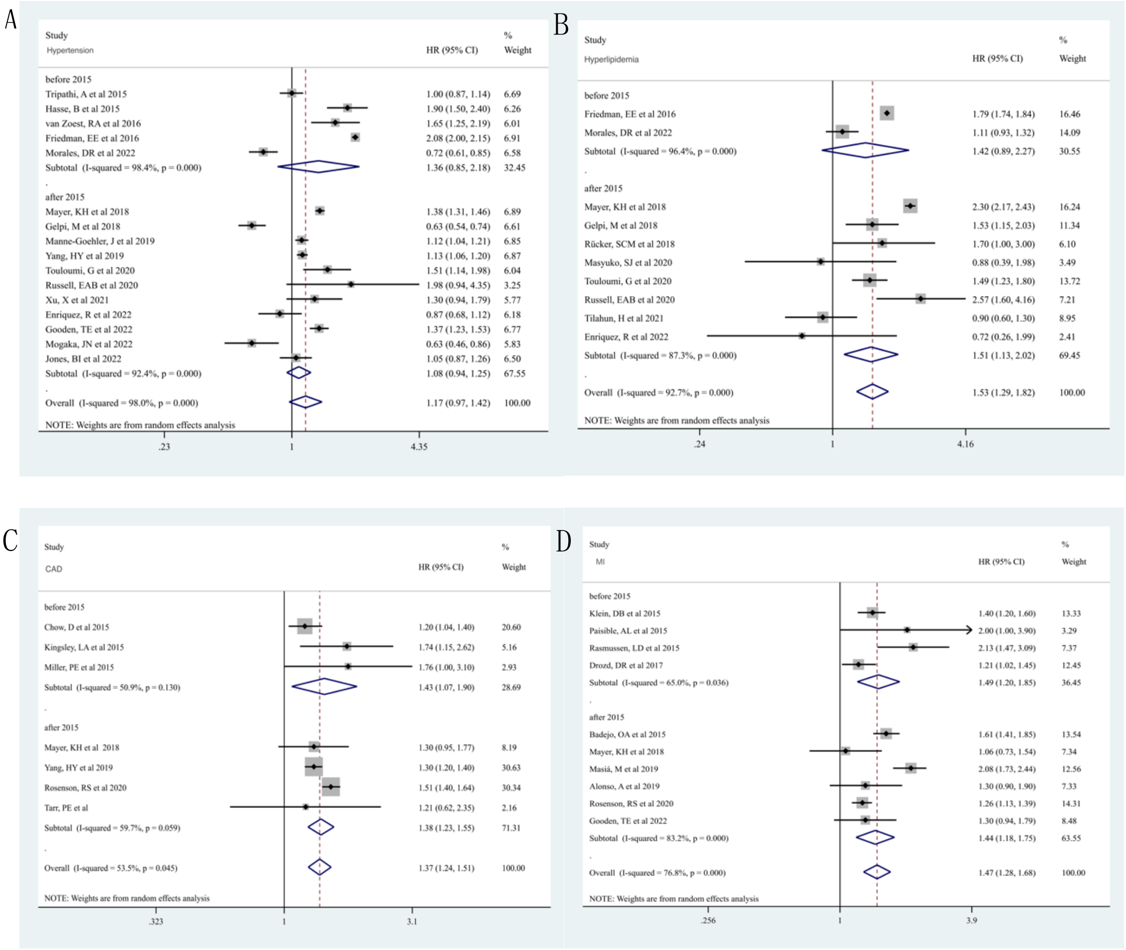


Abbreviations: CAD, coronary atherosclerosis disease; MI, myocardial infarction.

(A) Hypertension; (B) Hyperlipidemia; (C) CAD; (D) MI;

## Figure S5. Subgroup analysis based on the proportion of male.

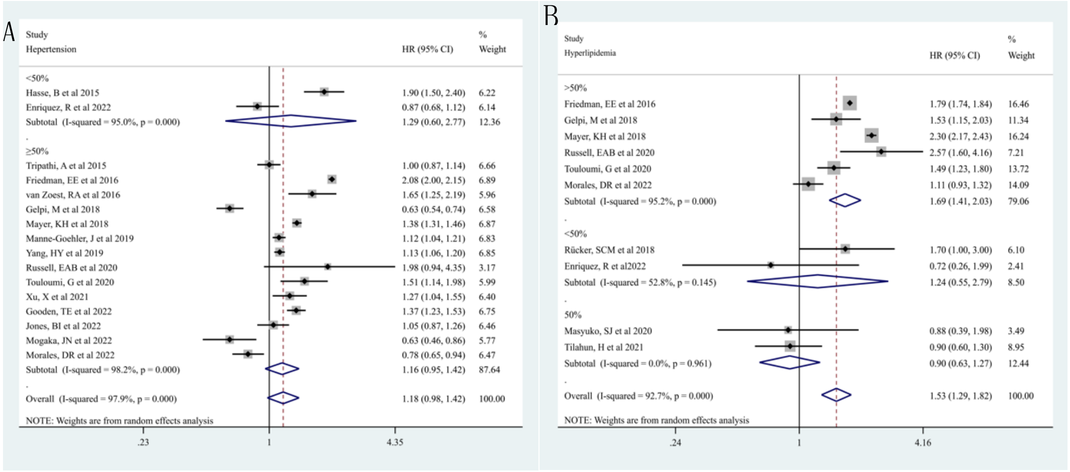


Abbreviations: CAD, coronary atherosclerosis disease; MI, myocardial infarction.

(A) Hypertension; (B) Hyperlipidemia;

## Figure S6. Subgroup analysis based on smoking.

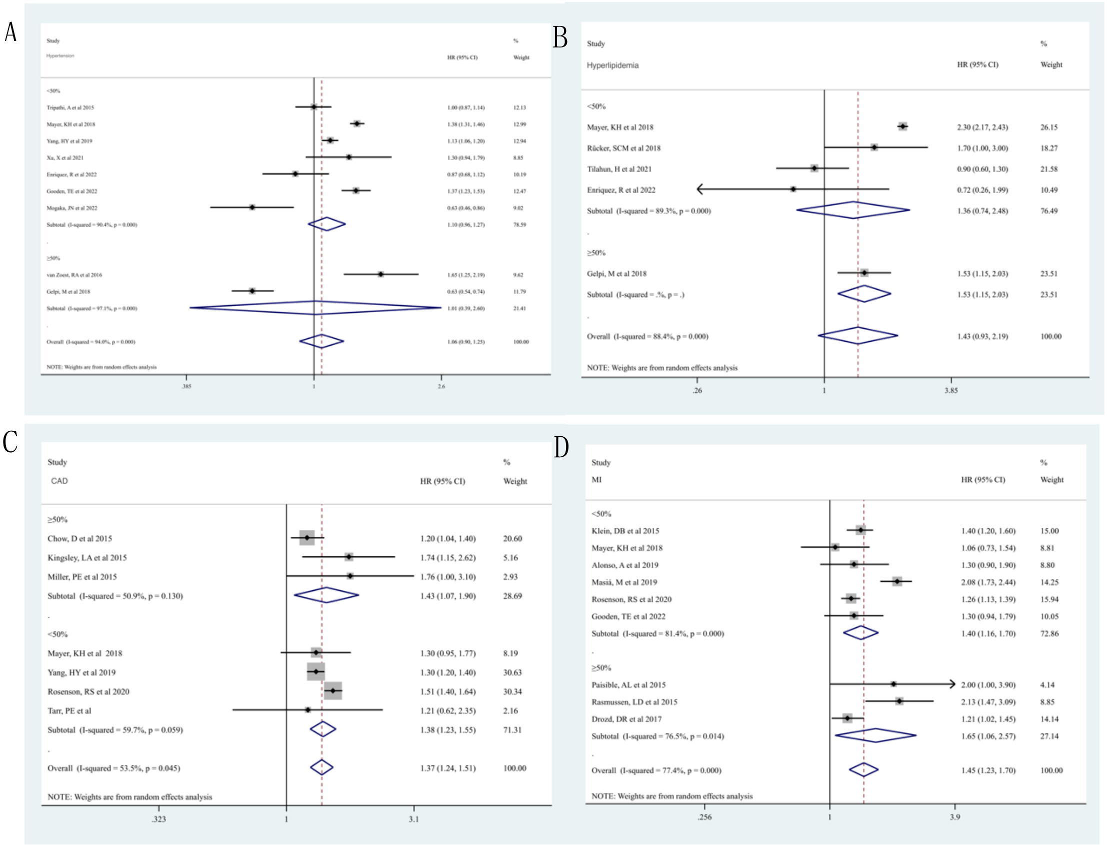


Abbreviations: CAD, coronary atherosclerosis disease; MI, myocardial infarction.

(A) Hypertension; (B) Hyperlipidemia; (C) CAD; (D) MI.

## Figure S7. Sensitivity analysis.

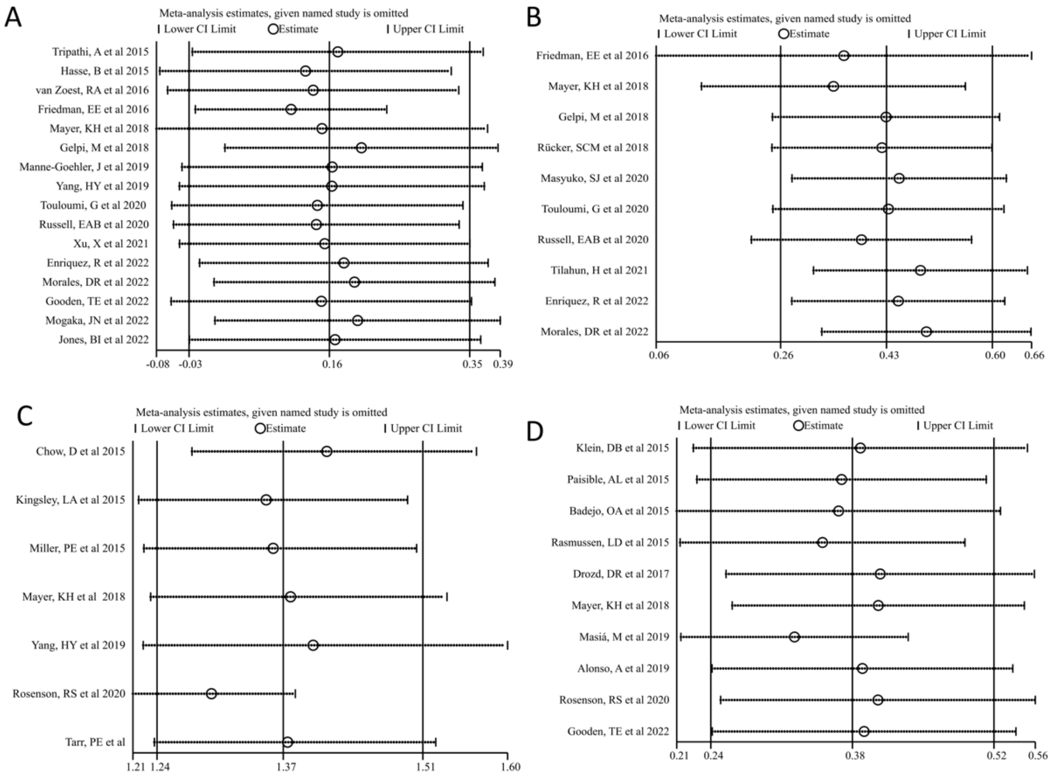


(A) Hypertension; (B) Hyperlipidemia; (C) CAD; (D) MI;
